# Optimizing usability of a mobile health intervention for Spanish-speaking Latinx people with HIV through user-centered design: a post-implementation study

**DOI:** 10.1093/jamiaopen/ooad083

**Published:** 2023-09-19

**Authors:** Kristen Petros De Guex, Tabor E Flickinger, Lisa Mayevsky, Hannah Zaveri, Michael Goncalves, Helen Reed, Lazaro Pesina, Rebecca Dillingham

**Affiliations:** Division of Infectious Diseases and International Health, Department of Medicine, University of Virginia, Charlottesville, VA 22908, United States; Division of General, Geriatric, Palliative and Hospital Medicine, Department of Medicine, University of Virginia, Charlottesville, VA 22908, United States; University of Virginia College of Arts and Sciences, Charlottesville, VA 22908, United States; University of Virginia College of Arts and Sciences, Charlottesville, VA 22908, United States; University of Virginia School of Medicine, Charlottesville, VA 22908, United States; Division of Infectious Diseases and International Health, Department of Medicine, University of Virginia, Charlottesville, VA 22908, United States; Nova Salud, Falls Church, VA 22044, United States; Division of Infectious Diseases and International Health, Department of Medicine, University of Virginia, Charlottesville, VA 22908, United States

**Keywords:** mobile health, digital health, Latinx, HIV/AIDS

## Abstract

**Objective:**

Latinx people comprise 30% of all new human immunodeficiency virus (HIV) infections in the United States and face many challenges to accessing and engaging with HIV care. To bridge these gaps in care, a Spanish-language mobile health (mHealth) intervention known as ConexionesPositivas (CP) was adapted from an established English-language platform called PositiveLinks (PL) to help improve engagement in care and reduce viral nonsuppression among its users. We aimed to determine how CP can address the challenges that Latinx people with HIV (PWH) in the United States face.

**Materials and methods:**

We conducted a post-implementation study of the CP mHealth platform, guided by principles of user-centered design. We enrolled 20 Spanish-speaking CP users in the study, who completed the previously validated System Usability Scale (SUS) and semistructured interviews. Interviews were transcribed and translated for analysis. We performed thematic coding of interview transcripts in Dedoose.

**Results:**

The SUS composite score was 75, which is within the range of good usability. Four categories of themes were identified in the interviews: client context, strengths of CP, barriers to use and dislikes, and suggestions to improve CP. Positive impacts included encouraging self-monitoring of medication adherence, mood and stress, connection to professional care, and development of a support system for PWH.

**Discussion:**

While CP is an effective and easy-to-use application, participants expressed a desire for improved personalization and interactivity, which will guide further iteration.

**Conclusion:**

This study highlights the importance of tailoring mHealth interventions to improve equity of access, especially for populations with limited English proficiency.

## Introduction

Although new statistics suggest progress in reducing the prevalence of human immunodeficiency virus (HIV) in the United States, there is also evidence of higher rates of infection among vulnerable groups.^1^ In particular, Latinx people living in the United States are diagnosed with HIV at disproportionate rates while concurrently experiencing health disparities due to a unique set of challenges.^2–4^ Social and economic factors contribute to a lower quality of care for Latinx people, including poverty, discrimination, and lack of access to health care,^5^ within a context of structural racism in the United States.^6^ Stigma related to HIV can be further exacerbated by stigma related to race/ethnicity, sexual orientation, and gender identity, which can also negatively impact access to care.^7^ Furthermore, language barriers and lower levels of formal education and health literacy contribute to misunderstanding the healthcare system and difficulty remaining virally suppressed or engaged in care.^8^

One emerging technology addressing these challenges for Latinx people is mobile health (mHealth). mHealth refers to the use of mobile, usually smart phones, to support health and healthcare. However, according to studies on consumer health technology interventions, there remain major gaps in mHealth efficacy. These investigations call for more tailored technology that meets patients’ needs and continuously engages patients to measure health outcomes.^9^ Many of these mHealth applications have been designed for English speakers; however, mHealth applications are less accessible to individuals with limited English proficiency and may further exacerbate linguistic inequities in healthcare.^10^ In addition, lack of access to smartphones and high-speed internet can be a barrier to participation in mHealth, particularly within marginalized communities, and contribute to health disparities.^11^

ConexionesPositivas (CP) is an mHealth intervention adapted from PositiveLinks (PL), an evidence-based intervention for people with HIV (PWH), that includes many features targeting the challenges faced by Spanish speakers in the United States.^3^^,^^12^^,^^13^ Some of these features include daily tracking of medication adherence, mood, and stress; appointment reminders; educational resources; laboratory results; and secure messaging with clinic staff and providers.^3^ CP was developed through translation and adaptation of PL, informed by feedback from the Spanish-speaking PWH who used the application during its pilot phase.

In this post-implementation study, we sought to further understand the context of challenges faced by Spanish-speaking PWH as well as their experiences with CP in order to further optimize this mHealth platform to promote improved access to, and engagement in HIV care for this priority population.

## Methods

### User-centered design framework

A user-centered design framework incorporates mHealth users in the design process.^3^ This approach can improve mHealth efficacy.^14^ User-centered design was implemented to assess the initial adaptation of PL for Spanish-speaking Latinx PWH. Therefore, in this follow-up study, the user-centered design approach was chosen to further improve CP for this priority population by investigating and then integrating participants’ ideas into the design process.^3^ CP is a self-monitoring application in which mood and stress check-ins and medication reminders are not monitored by the clinic, but are intended to empower patients to self-monitor and improve their health.

### Study design and participants

Individual interviews rather than focus groups were organized to avoid potential obstacles related to privacy among PWH. A semistructured interview guide was developed based on principles of user-centered design to assess usability of CP and on the team’s prior experience in evaluating usability of PL and other mHealth platforms.^3^

Participants included 20 Spanish-speaking Latinx CP users. This recruitment goal was set based on prior mHealth usability studies performed by our team^3^^,^^15^ and the literature on qualitative methods.^16^ Participants were required to be active CP users for at least 1 month, diagnosed with HIV, over 18 years old and native Spanish speakers. CP users can receive a monthly phone credit if they complete at least 48% of the self-monitoring queries to incentivize CP engagement and offset potential costs to participants of phone/data use. The participants in this follow-up study are comparable to the initial 2020 formative work adapting PL for Spanish-speaking Latinx PWH; 3 participants in this follow-up study were also involved in the 2020 formative phase.^3^ Participants included both monolingual Spanish speakers and individuals who are bilingual in English and Spanish. These participants were recruited by phone or in person at a Latinx-serving community-based organization (CBO) in the Southern US.

Interviews were conducted from February to March 2021. The protocol was approved by the University of Virginia Institutional Review Board for Health Sciences Research. All participants gave informed consent for the study.

### Data collection

Participants completed the 10-item System Usability Scale (SUS), a previously validated and reliable tool to assess usability of a variety of products, including mHealth apps.^17^ For this Spanish-speaking population, we used the Spanish version of SUS, which has high internal reliability.^18^ Semistructured interviews were carried out via phone and the SUS was administered by phone at the same time as the interviews. All interviews were conducted by a bilingual investigator with a graduate degree in Latin American studies. The interviews were then recorded, transcribed, and translated by said investigator, a native English speaker with near-native Spanish fluency certified in medical interpretation. A native Spanish speaker participated in transcription and translation, which were checked for fidelity.

Interviews assessed CP users’ experiences with the application. The development of the interview guide was informed by principles of the user-centered design framework, which emphasizes eliciting end-user experience with technology and seeking input directed at iterative improvement.^14^ For example, the interview guide included open-ended questions asking about participants’ experience with app usage, motivation to use the app, which features they liked most and why, which features they disliked or had trouble using and why, any change or impact on their HIV care related to the app, and any suggestions they had for improvement of the app.

The interviews ranged from 13 to 56 min, and all participants received a gift card as compensation.

### Analysis

After the interviews were conducted, transcripts were imported into Dedoose for analysis by 3 coders (Dedoose Version 8.0.35, web application for managing, analyzing, and presenting qualitative and mixed method research data [2018]. SocioCultural Research Consultants, LLC, Los Angeles, CA; www.dedoose.com). Each of the interviews was independently coded and then reviewed altogether to resolve any discrepancies until consensus was reached. The codebook was refined iteratively using constant comparisons methodology, until intercoder reliability and thematic saturation were achieved. The final codebook was used for all interviews. Thematic analysis was performed to identify what participants liked or disliked about the application and how this could be improved. Theme categories related to likes, dislikes, and suggestions for the app were informed by the user-centered design framework, intended to capture relevant aspects of participants’ app usage experience and input for improvement.^14^ Participants were not specifically asked about mental health, stigma, or preconceived aspects of their context, but these concepts were generated by participants in discussion of the app’s role in self-monitoring of mood and stress and their connection to care. Themes surrounding challenges to care and client context emerged inductively from analysis of the interviews and informed understanding of the barriers and strengths of the app discussed by the participants.

## Results

### Participants

Twenty participants were enrolled through a Latinx-serving CBO. We did not gather individual-level demographic information, in order to prioritize participants’ privacy. However, we can estimate from prior studies that our team has conducted in this population, in partnership with this CBO, that participants were likely 45% male, 45% female, 10% transgender; mean age 41 years, standard deviation 11.6 years; all born outside the United States, most commonly in Honduras, Mexico, and El Salvador.^3^

### System Usability Scale Survey

The SUS composite score for this cohort was 75, which is within the range of good usability.^17^^,^^18^ In a study for Spanish digital health applications, the mean SUS score was 68, which indicates that CP has above average usability compared to other mHealth applications.^19^ This usability score is also comparable to other studies that have used the SUS to evaluate mHealth applications related to HIV care and prevention.^20–22^ According to the SUS, most respondents stated that they would like to use CP frequently, the application is easy to use, has well-integrated features, is consistent, and that they felt confident using it. Responses to each item of the scale are reported in Table S1.

### Participant interviews

After coding the interviews, 4 categories of themes were identified: client context, strengths of CP, barriers to use and dislikes, and suggestions to improve CP. The results are summarized in 4 tables that feature themes within each category. For each table, percentages are reported as the number of code applications for each theme divided by the total code applications within that category. While it is important to consider the frequency associated with each theme, important themes mentioned once were included due to the theme’s potential to inform user-centered design. We also examined code presence across the interviews and found similar results for which themes were most prevalent. For example, mental health struggles and stigma were highly prevalent among code applications within client context and were also common across interviews, each mentioned at least once by 8 out of 20 participants (40%).

Client context (Table 1) encompassed participants’ challenges, values, and their strategies to manage their care effectively. Participants often discussed mental health struggles (19.4%), including depression or anxiety, as well as facing stigma due to their HIV diagnosis (12.6%). They also discussed life stressors (10.6%) that contribute to mental health challenges and lacking support systems due to nondisclosure of their HIV status (8.7%). In addition to these challenges, participants discussed personal strategies (10.6%) to overcome some of these adversities and support others living with HIV (4.8%). They also discussed how the application and engagement in care helped them find a new self-worth and meaning in their lives since their HIV diagnosis (1.9%).

**Table 1.** Themes in client context (*n* = 103) with frequency of occurrence, percentage of theme within category, and representative quotes.

Theme	Frequency (% within category)	Sample quote
Mental health	20 (19.4%)	“So, if the person has had many bouts of depression this month, what’s going on with that person? Let’s remember that sometimes, depression or anxiety or whatever, can also make a person reluctant to take their medication.”
Stigma	13 (12.6%)	“So, there should be respect for that. I don’t like to be called ‘sidoso.’ I don’t like to be called ‘contagious,’ or ‘infectious.’ They are words that are distasteful, stigmatizing, and I don’t like being called ‘patient,’ either. ‘Patient with HIV.’”
Lack of naming HIV	12 (11.6%)	“As I’ve already said, I’m new to this situation and I see that they have supported me.” “… I’ve only been dealing with this for two months.”
Personal strategies	11 (10.6%)	“I started doing meditation and I began telling them that it was working for me to do meditation every day. I was doing yoga and meditation, going outdoors, things to keep my mind occupied and not focus so much on the problem of the disease, and to look at the positive side of things.”
Life stressor	11(10.6%)	“So, in that way, it’s magnificent because sometimes you forget, and that’s true at times, because of all of the stress, or sometimes there’s so many things to do, or maybe the person forgot, or sometimes family problems make you forget about everything.”
Nondisclosure of HIV status	9 (8.7%)	“No one, because I don’t have support. No one in my family knows I have HIV.”
Misinformation about HIV	8 (7.7%)	“…there are people who don’t inform themselves and maybe they become depressed, they don’t want to eat or they only think about death. So, why? Because they’re misinformed.”
Isolation/loneliness	5 (4.8%)	“The thing is, so often, people begin handling this situation with depression and with solitude and with very little communication.” “Once you’re diagnosed with HIV, you isolate yourself a lot from others, or you feel like you’re not part of the same society.”
Desire to support others/advocacy	5 (4.8%)	“Running a group for LGBT people with HIV…I feel like I’m being useful for my community and the people allow me to share my knowledge, forming new leaders and forming people who really have that open-mindedness to be able to do new things.”
Immigrant experience	3 (2.9%)	“I’m not an open book myself, but I think that since I’m in a country that’s not my birthplace, and I’m receiving benefits—well, nowadays, I’m a citizen. But I’m in a country that’s not my birthplace and all it has done for me is give, give, give, in terms of my health—so, I don’t need to have so many qualms about whether they know or don’t know.”
Connection to clinical team	2 (1.9%)	“That woman is essential. And thank God, yes, she is very nice. I won’t lie to you. She has helped me very much. She was my first contact. And look where I’ve ended up now.”
Self-worth/meaning in life	2 (1.9%)	“It made me feel more connected to myself in that way and today I’m happy, you could say, to be HIV positive. I know it’s strange to hear, but I take pride in that.”
Comorbidities	2 (1.9%)	“I didn’t know that my syphilis was positive and they took my blood. They gave me a hepatitis test, which a lot of people don’t do.”

CP’s strengths (Table 2) were mentioned 125 times by the 20 participants. Of these strengths, access to information/education (19.2%), privacy (15%), and connection to professional care (12.7%) were the most frequently mentioned. Due to participants’ perception of CP’s positive impact, the impact of specific application features is included in the strengths section. Of the 51 times that a positive impact of the application was mentioned, 47.1% expressed a positive impact on medication adherence, 41.1% on mood/stress, and 11.8% on client-provider communication. Looking at code presence across interviews, privacy as a strength was most common, mentioned at least once by 19 out of 20 participants (95%), followed by ease of use, mentioned by 16 (80%).

**Table 2.** Themes in strengths of CP (*n* = 166) with frequency of occurrence, percentage of theme within category, and representative quotes.

Theme	Frequency (% within category)	Sample quote
Access to information/education	32 (19.2%)	“You learn about things that you did not have the answers to before.”
Privacy	25 (15%)	“No, the privacy part is normal. I have no problem with it, since no one knows who you are. So, that’s the least of my worries.”
Connection to professional care	21 (12.7%)	“The [feature] I use the most is the one with all of my providers’ contacts…I can always get in touch with my providers and I think it’s very, very good because it’s a safe place to go look up contacts.”
Ease of use	21 (12.7%)	“It’s extremely easy. It’s very user friendly. Logging in is very secure. It’s really fast. Navigating around the app is very user friendly, very easy. Everything is really easy.”
Support system	18 (10.8%)	“Before, we were practically hidden away from the world because here, among ourselves we put up with one another, because we thought we were the only ones with HIV or with the condition, and now that we have a community, and we have a group, and we have support and all of that, it’s different.”
Motivational messages	10 (6.0%)	“They’re very creative because they help you—if you have a bad day, it tells you, well, ‘Do your best,’ or like, ‘I hope you feel better,’ or things like that.”
Feeling valued/more confidence	10 (6.0%)	“I feel more confident in myself. That they value me more. Do you understand? I feel stronger, or something like that. Stronger, yes.”
Sharing experiences	9 (5.4%)	“I feel confident because only people living with the same condition are there. There’s not going to be a person who is going to—who is going to criticize you, ‘Oh, he has this!’ So, it’s a community of free expression.”
Positive change	9 (5.4%)	“It made me feel more connected to myself in that way and today I’m happy, you could say, to be HIV positive. I know it’s strange to hear, but I take pride in that.”
Phone credit incentive	7 (4.2%)	“In the sense that the app provides us with a sort of financial assistance. That’s why I do it, why we do it—respond to those questions day to day, to reach a percentage that determines whether we receive the benefit or not.”
Technology support	3 (1.8%)	“But it was hard for me at the beginning. There were a lot of technical failures…[PL staff name] would tell me over Skype what I had to do. She would send me a screenshot of everything I had to do, and now it works.”
Virtual alternative	1 (0.6%)	“‘I don’t have time.’ I told him, ‘I can’t make it out to where you all are.’ ‘No,’ he says. ‘It’s over the phone,’ he tells me. ‘Oh, okay. If it’s over the phone, I can,’ I told him. ‘I gladly accept,’ and everything.”
Positive impacts of the application (*n* = 51)		
Impact on medication adherence	24 (47.1%)	“And honestly, it helps a lot because sometimes, as I’m telling you, you’re in a rush with dinner, the kids and you sometimes forget about your pill, but with the app, you see there’s always a reminder there and that it’s time to take it.”
Impact of mood/stress check-in	21 (41.1%)	“Mostly it helps me think about how I feel or how my stress is, and during that time I really analyze my day, each thing, and I really see how I’m doing. Before, I didn’t do that; before, it was more like, today is just a day, and the day has to go by, however it may go. But today, now I can focus more on thinking, ‘What can I do better?’ every day.”
Impact on client-provider communication	6 (11.8%)	“Now I can use the application to communicate with them directly. Better. Yes, much better. Yes, because I send messages to communicate with [provider name] or with [provider name] or with [provider name], who is my therapist.”

Barriers to use and dislikes regarding CP (Table 3) were mentioned 73 times. Of these barriers, privacy (14.9%), low educational level/general literacy (14.9%), and lack of personal connection (13.8%) were the most frequently mentioned. Looking at code presence across interviews, privacy as a barrier and password/log-in difficulty were most common, each mentioned at least once by 8 out of 20 participants (40%).

**Table 3.** Themes in barriers to use and dislikes (*n* = 87) with frequency of occurrence, percentage of theme within category, and representative quotes.

Theme	Frequency (% within category)	Sample quote
Privacy (barrier)	13 (14.9%)	“But if there were a way to send personal messages, it would be much better. But I know that because of privacy, you can’t because we’re not allowed to know who is who. Let’s say that I have some friends there, but I don’t know which name they use. No one knows who is who, but if you found out, you could chat, send them messages.”
Low educational level/general literacy	13 (14.9%)	“I don’t know how to read very much, you know. I don’t know how to write very well. That’s why I just like to listen or read what they say, because since I can barely write, that’s why I’m telling you that I like listening to everyone else’s experiences more.”
Lack of personal connection	12 (13.8%)	“But there’s no interaction with my peers; there’s no interaction with the—I mean, I know there are people available there. I don’t feel that trust because I don’t know who they are; I don’t know them.”
Password login	9 (10.3%)	“The part where you always have to put in your password (laughter) every time you’re going to log in. I’m not sure if everyone has to do that, but whenever I want to log in, I always have to enter the password to log in.”
Competing priorities	7 (8.0%)	“I’d like to know more about, I mean, I don’t have enough time to be on the app a lot. It’s only when I see the questions [check-in’s] that I log in and stay there for a little bit looking at everything. Otherwise, I don’t. Because of work.”
Lack of trust	6 (6.9%)	“…the people who are there, like my providers, I don’t know some of them. I don’t have the confidence to speak with them and take initiative.”
Low technology literacy	6 (6.9%)	“But it was hard for me at the beginning. There were a lot of technical failures. I couldn’t figure out how to make it work. The password would fail me, the user name, and everything else.”
App not challenging enough	5 (5.7%)	“Of course, it’s way too basic. So, it’s—if you didn’t know something, I think you’d have to be very ignorant.”
Repetitiveness	5 (5.7%)	“I mean, sometimes, like I’m telling you, I forget because it’s, like, boring to log in, log in every day, doing the same thing, over and over and over; right? Like, at the beginning, I would do it more frequently, I was more motivated to, but as time passed, like, it becomes a bit monotonous.”
Stigma (barrier)	4 (4.6%)	“If I see that there are people around, like, you sometimes feel inferior. I don’t know why. They might be curious about it and, I don’t know.”
Concern for CMB negativity	4 (4.6%)	“I think it would have to be some comments that aren’t so good or are too depressing and, really, you don’t—sometimes I don’t even look at the comments.”
Lack of awareness of features	2 (2.3%)	“It was only at the beginning that I didn’t know; I thought that it was only for keeping track of my medicine. Later I realized that there were other features.”
Language barrier	1 (1.1%)	“But there are some applications that are translated to English, it seems—some, not all. You know that for an American, well, they already know what it says, but country bumpkins [offensive], as they say, well, they don’t understand much English.”

In addition to describing the barriers or dislikes of CP, participants offered suggestions on how to improve the application (Table 4). In general, participants requested more information about HIV and stigma (11.8%) and more interactive application elements (11.8%). They also called for more personalization within CP; specifically, users wanted to manually set CP’s medication or mood/stress check-in times (8.5%). Participants also requested more personal connection (6.5%) through different mediums, such as directly and consistently incorporating PWH in CP’s development process, reducing anonymity to enable face-to-face interaction, and enabling direct messaging opportunities. Looking at code presence across interviews, more interactive elements was the most common suggestion, mentioned at least once by 10 out of 20 participants (50%), followed by more information on HIV/stigma, mentioned by 9 (45%).

**Table 4.** Themes in suggestions (*n* = 153) with frequency of occurrence, percentage of theme within category, and representative quotes.

Theme	Frequency (% within category)	Sample quote
More information on HIV/stigma	18 (11.8%)	“I know the application already has a lot of informative content, but sometimes—well, for me, personally, I would like it if it had recent summaries about research studies that are being done—how do I say—recent studies that they are doing about the disease.”
More interactive elements	18 (11.8%)	“I think it would be good if they asked us questions, like a mini game to make us participate a bit more. It would be a little hard to do, but yes—like a mini introduction or something—a topic that gets us talking would be good, to think and see what different reasoning or ideology each one of us has. “
More personalization	13 (8.5%)	“Like, “You have improved your adherence.” What can we do in the event that it’s not working, or it’s not…how would you say it? “You have had a very depressed week,” I mean, “How can we help you?” More personalized, maybe, is how I see it.”
More personal connection	10 (6.5%)	“You can’t say or type, “Look, chat with me in private in case you need guidance on how to handle yourself, how to manage your life after you’re in a system, on a medication regimen,” or like… You shouldn’t be left adrift just answering yes or no, or a happy face or a sad face; rather, it should be dynamic”
Keeping app updated	9 (5.9%)	“You know, the resources section is very good, it appealed to me because it has very important information in it, very complete. But that’s it. I mean, I already read it, I already finished it. So, it would be good to maybe update it a little bit.”
More visuals/audio	9 (5.9%)	“It’s well-organized and everything but, as I’m telling you, it’s always the visuals. And they never change the colors or anything. Sometimes they need to—even if it’s sending a subliminal message that gives you energy, that gives you a desire to keep living.”
More challenging quiz questions	8 (5.2%)	“They should be questions that are a bit more complicated; questions that make you think and say, “What’s this about?”
Outreach from program staff	8 (5.2%)	“I think there should be, or what’s missing is that they should use the information about people’s mood. I’m not saying they’re going to solve it, but at least seek out assistance or help that could, in some way, improve their stress or mood, which is what the app asks about most, right?”
Spanish resources/notification/other features	3 (2%)	“Well, for example, when—the only thing is when they make an appointment for me, they put it in English. So, you know that not everyone knows English, you can’t even read it.”
Offer access to mental health emergency line	2 (1.3%)	“…the app can be an extremely helpful tool in other cases, and that it could be worse, or it could end in a fatality, right? [I’d like to be able to] find there, aside from resources where I can look for help, people who, in that moment, could respond and I could feel confident enough to be able to talk to without being judged.”

## Discussion

This study shows that the mHealth intervention, CP, is well-adapted to meet the needs of a Spanish-speaking Latinx population of PWH with good usability, multiple perceived strengths, and a reported positive impact. Following the framework of user-centered design, we identified ways CP can be further improved to better meet clients’ needs and encourage engagement with the application. This post-implementation study represents a next step after the initial formative work performed to adapt the PL intervention to a new population, eliciting users’ experiences and input to support iterative development. This study fills a gap in the field of mHealth that generally focuses on interventions for English-speaking populations of higher literacy and socio-economic status.^23^ There is an ongoing need to improve equity in access to the benefits of mHealth, which can improve chronic disease management, but only when optimized to reach the intended users.^23^

Using the previously validated SUS, this study demonstrated good usability of CP and this quantitative data aligned with participants’ perceptions expressed in the interviews. Ease of use was cited as one of the strengths of CP. However, some users struggled to adjust to the application at first due to low technology literacy. These participants were able to access the necessary assistance to become comfortable with the application with assistance from CBO-based team members. Although participants with low literacy could use the text-to-voice or voice-to-text capabilities of their phones if needed, we did not assess how often they did so in using text-based features like the community message board.

Many of CP’s strengths corresponded to concerns expressed by participants in describing challenges to engagement in HIV care, suggesting that CP may help overcome or address these challenges. For example, one of the strengths frequently highlighted was CP’s access to information and education, which may combat the misinformation about HIV that concerned some participants. Participants also appreciated CP’s protection of their HIV-positive status or privacy. In prioritizing privacy, CP respects patients’ concerns about wanting to maintain nondisclosure of HIV status. Nondisclosure of HIV status among our participants was driven by internal and external stigma, as demonstrated by their lack of naming of HIV and desire to be seen as more than a patient with HIV.

Participants also described positive aspects of client context, which can facilitate engagement in HIV care, including the importance of connection to their clinical team, desire to support others, and a sense of self-worth. In addition to addressing barriers to care, CP can be used to enhance facilitators. Participants expressed that the application effectively connects them to professional care and can enhance client-provider communication. Consequently, CP users can be empowered to communicate concerns or questions with their providers. Another key strength was the application’s community. Participants highlighted that they appreciated the support system within CP, felt more valued and confident, and appreciated the opportunity to share their experiences with other PWH. They also highlighted the positive impact of CP’s motivational messaging. Overall, CP may provide a strategy to help mitigate the loneliness and isolation that some participants reported feeling due to their HIV diagnosis. Mental health and stigma were the 2 most frequently mentioned aspects of client context in participants’ interviews, which emphasizes the importance of CP features designed to address these concerns. CP offers a space where PWH can be themselves and uplift each other during challenging times by connecting them to providers and a community of PWH.

It is important to consider that some participants specifically noted they appreciate the phone credit offered to CP users. This was a monthly credit applied to users’ phone bills if their usage met a frequency threshold, in order to offset costs to clients from use of their phones and data. The phone credit may motivate some people who otherwise would not use the application to engage with CP. As a result, there is the potential that people could be logging into the application, randomly selecting responses to meet the minimum usage requirements, and then logging out. Nonetheless, only a small percentage of users commented on the application’s phone credit incentive for usage. Potential benefits of financial incentives to mHealth participation should also be considered, as they may reduce barriers and improve equitable access to mHealth for populations with socio-economic obstacles to smartphone use.^24^

CP is designed for PWH to engage in self-monitoring. Study participants expressed a perceived positive impact of CP on medication adherence and on their mood/stress. It is notable that they were specifically asked about CP’s impact on mood and stress and medication adherence in the study interview guide. In contrast, the client-provider communication was not consistently prompted and may have been mentioned less frequently as a result. Respondents who did not report a positive impact of CP stated its effects as neutral with none giving a negative impact. Throughout the open-ended interviews, it was clear that some participants were not aware of CP’s purpose as a self-monitoring application. This was evident from the participants who desired CP’s staff to intervene through text or a phone call when they experienced a day with poor mood or stress. In addition, some participants called for more outreach from CP’s staff. As a result, in future application development, it is crucial to consider client understanding of the purpose of each feature and the optimal balance between self-monitoring (the focus of the check-in queries) and outreach or connection with CP staff (the focus of secure messaging features).

Participants highlighted barriers to CP usage and suggestions that can inform user-centered design moving forward. The team organized these suggestions into overarching topic areas, including those related to education/training in app use, app design, and resources that could be accessed through the app. The team also assessed feasibility of suggestions and short-term versus long-term goals for iteration. For example, participants emphasized that the application would be difficult to navigate for those with a low educational level/general literacy. A potential way to address this in the short-term would be integrating more visuals and audio so that information on CP is more accessible to those with low literacy. Participants also called for CP to update its resources more frequently and include emergency mental health resources, such as a hotline phone number. This study showed that users rely on the application for important resources, so it is important to ensure they are consistently updated.

With regard to longer-term improvements to the application, participants expressed frustration with the application’s lack of personal connection. Many wanted one-on-one interaction, and some participants expressed that the limited connection between users leads to a general lack of trust among participants. Potentially, there could be opportunities for users to engage in real-time virtual groups or online discussions to connect more personally. CP’s developers will need to consider balancing increasing personal connection while maintaining participant confidentiality. For clients who reside in areas with a high density of Spanish speakers, in-person connections may play a more prominent role in their social support than virtual interaction. CP can still be used to inform clients of in-person opportunities taking place outside the application. Some participants also expressed that the application’s quiz questions were not challenging enough for them. This appeared to be an issue specifically for participants who had long-term experience with HIV and were already well informed, in contrast to other participants who were more recently diagnosed and still learning key information about living with HIV. Therefore, CP could better implement different levels of quiz questions to accommodate their users’ varied understanding of HIV. This is especially important to address some users’ concerns over the repetitive interactive elements and ensure that these quizzes engage users, so they are motivated to use CP each day.

Limitations of the study include that participants were only recruited from a single site in Virginia. As a result, transferability may be limited because Spanish-speaking PWH in different contexts or backgrounds may not share the same experience if they implemented the application. In addition, for this investigation, participant demographics were excluded to protect their privacy; as a result, there are potential gaps in our understanding of possible demographic factors driving challenges to CP usage. In addition, selection bias may be present if individuals who are motivated to engage in a research study are more likely to be active or more engaged CP users or have higher technology literacy than those who did not participate.

Overall, next steps for CP development include optimization of Spanish-language training videos, expansion of quiz questions (such as through crowd-sourcing), and continuous updates to resources. The English-language PL application also undergoes periodic updates, including short-term additions such as a “like” option for CMB posts to encourage interaction and long-term plans to refresh color palettes and aesthetics. Our team is conducting an ongoing effort to adapt PL and its related tutorials and support materials informed by the Web Content Accessibility Guidelines (WCAG) which seek to improve accessibility of online materials for people with diverse needs and disabilities.^25^ Examples of these guidelines include providing alternative ways to perceive content, alternative means of input to interact with content, ease of navigation through content, captioning, color contrast, and other strategies to accommodate users’ needs and preferences. It will be important for updates to be adapted for CP, as well as PL, and for Spanish-speaking PWH to be included in iterative development processes. While use of the check-ins by CP clients has been particularly popular, there are opportunities to increase engagement with other PWH and with providers. The CMB includes staff posting to keep the CP community informed about announcements and mental health support services, which facilitates interaction outside the app as clients connect with each other for in-person support. Encouraging provider use of the app would be needed for the secure messaging feature to reach its potential in enhancing client-provider communication.

In addition to further iteration of the CP mHealth intervention, participants’ experiences may also be used to inform broader issues of health policy. Client context themes emphasize ongoing needs to address access to mental health services and efforts to reduce stigma related to HIV and minority/immigrant status. This study also highlights the need for policy that improves equitable access to the benefits of mHealth for populations often overlooked in technology development, included those with limited English proficiency.

## Conclusion

There is an identifiable gap in the use and tailoring of mHealth applications for Latinx PWH. This study identifies CP as an effective, easy-to-use mHealth application among Spanish-speaking PWH. The user-centered process employed in the study identified the strengths, barriers, and suggestions for the application, which will inform further iterations of the app to continue its positive impacts on the participants. In particular, positive impacts include encouraging self-monitoring of medication adherence, mood and stress, connection to professional care, and development of a support system for PWH. Although other major strengths in the application were identified in key design features such as privacy and resources, participants seek further information, interactive features, and more personalization of the application. With continued improvements, CP can optimize its user experience through increased accessibility and resources. Future research can identify how these changes impact usability and over time, how mHealth participation can enhance engagement in care and clinical outcomes.

## Supplementary Material

ooad083_Supplementary_Data

## Data Availability

The data underlying this article cannot be shared publicly due to a need to protect the privacy of individuals that participated in the study. The data will be shared on reasonable request to the corresponding author.
