# Supplementary material for: Optimizing usability of a mobile health intervention for Spanish-speaking Latinx people with HIV through user-centered design: a post-implementation study
Source: JAMIA Open. 2023 Sep 19;6(3):ooad083. doi: 10.1093/jamiaopen/ooad083 (PMC10508965; doi:10.1093/jamiaopen/ooad083)
Supplement: ooad083_Supplementary_Data [file ooad083_supplementary_data.doc]

Supplemental Table 1: CP Member Usability Assessment

| # | Item | Strongly Disagree | Disagree | Neutral | Agree | Strongly Agree |
| --- | --- | --- | --- | --- | --- | --- |
| 1 | I think that I would like to use this app frequently. | 1 |  |  | 10 | 9 |
| 2 | I find this app unnecessarily complex. | 4 | 14 | 1 |  | 1 |
| 3 | I think this app is easy to use. |  | 1 |  | 12 | 7 |
| 4 | I think that I would need assistance to be able to use this app. | 2 | 14 | 1 | 3 |  |
| 5 | I find the various functions in this app were well integrated. |  | 2 |  | 14 | 4 |
| 6 | I think there is too much inconsistency in this app. |  | 16 | 1 | 2 | 1 |
| 7 | I would imagine that most people would learn to use this app very quickly. |  |  | 1 | 15 | 4 |
| 8 | I find this app very cumbersome/awkward to use. | 5 | 14 |  | 1 |  |
| 9 | I feel very confident using this app. |  |  | 1 | 12 | 7 |
| 10 | I needed to learn a lot of things before I could get going with this app. | 2 | 12 | 3 | 3 |  |
